# Incidence of Thyroid Cancer in Italian Contaminated Sites

**DOI:** 10.3390/ijerph18010191

**Published:** 2020-12-29

**Authors:** Marta Benedetti, Amerigo Zona, Paolo Contiero, Eleonora D’Armiento, Ivano Iavarone, AIRTUM Working Group

**Affiliations:** 1 Department of Environment and Health, Istituto Superiore di Sanità, 00161 Rome, Italy; amerigo.zona@iss.it (A.Z.); ivano.iavarone@iss.it (I.I.); 2 Environmental Epidemiology Unit, Fondazione IRCCS Istituto Nazionale dei Tumori, 20133 Milan, Italy; paolo.contiero@istitutotumori.mi.it; 3 Internal Medicine Unit, Department of Medico-Surgical Sciences and Biotechnologies, ICOT Hospital, University of Rome, 04100 Latina, Italy; eleonora.darmiento@fastwebnet.it

**Keywords:** thyroid cancer, incidence, endocrine disruptors, environmental exposure

## Abstract

Some human literature suggests a possible role of endocrine disruptors (EDs) exposure in thyroid cancer (TC) development. We investigated TC incidence in selected Italian National Priority Contaminated Sites (NPCS) with documented presence of EDs considered thyroid carcinogens. Adjusted Standardized Incidence Ratios (SIRs), with their 90% confidence intervals, were computed by gender, and age-specific groups (aged 15–39 years, and 40 years or over) for each NPCS in the period 2006 to 2013. In the age group of 15–39 years, a significant excess of TC risk was found in two NPCSs in males; non-significant excess risks were observed in four NPCSs in males, and in five in females. In the age group of 40 years and over, significant excess risks were found in six NPCSs in males and in seven NPCSs in females; non-significant excess risks were identified in two NPCSs in males and females. The findings of several excesses in incidence, mainly observed in adults aged 40 years or over, are suggestive of a possible adverse effect associated with residence in NPCSs, even if a role of other factors cannot be excluded, due to the adoption of an ecological study design. Future analytical studies are needed to clarify if EDs are a TC risk factor for individuals living in NPCSs.

## 1. Introduction

Thyroid cancer (TC) represents the most common endocrine malignancy accounting for roughly 1–3% of all human tumors. It is two to four times more frequent in women than in men, being the second most frequent tumor in the age group 0–49, and the fifth most frequent in the age group 50–69. TC is one of the most common malignancies in adolescent and young adults, aged 15–39 years, the median age at diagnosis being lower than that for most other types of cancer. In children, especially before 10 years of age, the occurrence of TC is very rare [1,2].

TC has a wide spectrum of histotypes. The most frequent being papillary (PTC) and follicular (FTC), known as well-differentiated TC, representing about 85–90% and 5–10% of all TC, respectively. Other less common forms of TC are the poorly differentiated TCs, accounting for less than 5% of cases, such as medullary TC, representing approximately 2% of TC, the rare Hurtle cell carcinoma, and the extremely rare and aggressive anaplastic TC, that occurs almost exclusively in older adults. Less common TCs are thyroid lymphomas, thyroid sarcomas, or other rare tumors [3].

In the last two decades TC incidence, mostly papillary, has increased worldwide more than any other cancer, especially in countries characterized with a high income, Italy included [4]. Some authors have attributed the increased incidence of TC to the improved ability to diagnose malignant transformation in small non-palpable thyroid nodules [5,6]. Others have argued that the increase cannot solely be explained by overdiagnosis of microcarcinoma, as there is also an increase in detection of large tumors and advanced-stage diseases [5,7,8,9]. Moreover, overdiagnosis is less likely to occur in children than adults because thyroid nodule screening would be rarely required for this age group and medical imaging of the neck would not be usually performed for other clinical purposes [9,10]. In addition, a rise simply caused by over-detection would not easily account for the increased incidence observed in the developing world, where imaging techniques are less affordable [10,11].

The thyroid gland is a histologically and functionally complex organ, which is very vulnerable to toxicants. Recent evidence suggests that environmental factors and lifestyle might have contributed to the increased incidence of TC, particularly the environmental exposure to some pollutants present in several environmental compartments [12,13]. Numerous studies have shown that some environmental chemicals (named endocrine disruptors, EDs) have the ability to disrupt metabolism of thyroid hormones at nearly every step, but much less is known about their relationships with TC [14,15]. In recent years, EDs have gained interest in their possible role in human thyroid carcinogenesis [16].

According to evaluations from some scientific institutions and advisory committees (World Health Organization and theUnited Nation Environment Programme (2013) [17], European Commission (2012) [18], and European Environment Agency (2012) [19]), the exposure to some EDs (dioxins (PCDDs), particularly 2,3,7,8-tetrachlorodibenzo-p-dioxin (TCDD or dioxin), polychlorinated biphenyls (PCBs), pesticides, and some solvents) might be a risk factor in the development of TC. Furthermore, other studies suggest that, in addition to the above-mentioned substances, chemicals and some heavy metals with an endocrine disrupting activity (polychlorinated dibenzo-p-furans (PCDFs), polybrominated diphenyl ethers (PBDEs), bisphenol A (BPA), flame retardants, phthalates, cadmium (Cd), and lead (Pb)) might also play a role in the etiopathogenesis of TC [20,21,22,23,24,25,26,27,28,29,30,31,32]. Regarding the possible role of heavy metals in promoting thyroid tumorigenesis, Malandrino et al. (2020) reports a higher risk of papillary TC in residents in the volcanic area of Mount Etna (Catania province, Southern Italy) [33]. High urine Cd, Pb, and mercury (Hg) levels were found in residents of Catania province; high levels of these elements were also found in the drinking water of the Mount Etna aquifer, which provides water to most of the residents in the Catania province. Uetani et al. (2006) [34], in a study aimed to compare organ Cd concentrations of people living in some Japanese Cd polluted areas, found that the thyroid gland cadmium concentration was three times higher in individuals living in polluted areas when compared to individuals residing in non-polluted areas.

Numerous chemicals and toxic by-products (e.g., TCDD, furans, PCBs, cadmium, lead, and solvents) are released or used in various industrial processes. However, few studies have been carried out to evaluate the occurrence of TC in the general population living in proximity to industrial contaminated sites.

One of the first published papers on this issue was by Grimalt et al. (1994) [35]. They observed a significant excess of TC mortality in men residing in an area with high levels of hexachlorobenzene (HCB). This was detected in air and blood sera from volunteers of a village located close to an organochlorinated-compounds plant (Flix, Catalonia, Spain). Fernandez–Navarro et al. (2012) investigated cancer-related mortality in populations residing in towns next to some Spanish mining facilities, from 1997 to 2006 [36]. Population exposure to mining facilities (hypostasized as dioxins, mercury, arsenic, lead, cadmium, polycyclic aromatic hydrocarbons (PAHs), and numerous others) was estimated based on distance from town of residence to pollution source (5 km radius). The authors observed that residing close to underground coal mining was related to an excess of TC, both in males and females, pointing to potential environmental exposure. Kloczko (2016) carried out an exploratory analysis on the risk of TC and residential proximity to industrial manufacturing facilities in a population-based case-control study in Connecticut from 1990 to 2009 [37]. The risk estimates were corrected for age, BMI, gender, prior alcohol use, family history of thyroid disease, family history of TC, and previous exposure to diagnostic radiation. The study results showed some non-significant excesses of TC among people who lived within 2 or 5 km from chemical product manufacturing facilities. Benedetti et al. (2017) investigated the incidence of breast (females), prostate, testicular, and thyroid cancer from 1996 to 2005 in 14 Italian National Priority Contaminated Sites (NPCS) with a documented presence of EDs [38]. Significantly increased SIRs were found for TC in four NPCSs in both genders, and non-significant increases in SIRs in two NPCSs in males and in one NPCS in females. Fei et al. (2018) analyzed the spatiotemporal pattern of TC from 2008 to 2012 in Hangzhou, a highly urbanized area with the highest TC incidence in China [39]. Three main factors were strongly and positively associated with female and male TC incidence: the emission of Chemical Oxygen Demand (COD; proxy for organic pollutants), industrial density and percentage of building area. For both genders there were significantly higher clusters in the north-eastern urban area with a two-fold increased risk for TC, and significantly lower clusters were mainly detected in the south-western rural areas (with a decrease in relative risk). The authors argue that the intensive industrial activities lead to high pollution levels, organic pollutants, such as PCBs and PBDEs that might disturb thyroid function and indirectly lead to a higher TC risk. Arias–Ortiz et al. (2018) carried out an ecologic study by performing a spatial analysis of TC incidence in women living in the proximity of a complex industrial setting in Colombia [40]. That industrial setting had released into the atmosphere pollutants identified as TC promoters, such as dioxins and furans, dichloromethane, lead and copper. TC incidence was significantly associated with residing near industrial facilities releasing dichloromethane or PCDDs and PCDFs. A specific cluster was observed close to an industrial facility that had released dichloromethane and tetrachloroethylene. In the last update of the SENTIERI Project (2019) [41], whose purpose was to study the health status of the population living near NPCSs, the cancer incidence in children (aged 0–14 years), adolescents (aged 5–19 years), and young adults (aged 20–29 years) was analyzed for the first time in the period 2006–2013. A statistically significant excess of TC was found only in NPCSs with documented presence of EDs: Taranto (25 female cases, aged 20–29 years, SIRs = 1.8; C.I. 90%: 1.3–2.6), Sassuolo–Scandiano (four male cases, aged 25–29years, SIRs =3.6; C.I. 90%: 1.2–8.1), and Fidenza (nine female cases, aged 0–29 years, SIRs = 2.9; C.I 0.90%: 1.5–5.1). This study, in the eight-year period, identified only two TC in children (0–14 years) in all 28 NPCSs (0.8% of all pediatric malignant tumors in this age class). This very low occurrence did not allow for the making of any informative analyses in NPCSs characterized by presence of EDs. However, it is worthwhile to notice that a recent paper (Bernier et al., 2019) showed a significant increase in rates of differentiated thyroid cancer over time in children aged 10–19 years in the United States [1]. The authors stated that this increase cannot be entirely due to improved medical surveillance during childhood and that environmental and other factors might partially be responsible for rising the rates.

Although recent research has focused particularly on exposure to chemicals and heavy metals with endocrine disrupting activity, other factors such as radiation exposure, iodine deficiency, gender, genetics, and obesity may also raise the risk of developing TC. Exposure to ionizing radiation, from incidents such as those of nuclear disasters, appears to be the most important contributor for papillary thyroid carcinoma with strong supporting evidence [42,43]. Chronic iodine deficiency is firmly established as a risk factor for goiter and follicular thyroid carcinoma [44]. Differentiated TC is markedly more common in women than men: upon the onset of puberty the incidence increases only in females and declines after menopause. The highest female-to-male ratio being recorded during the reproductive period suggests that estrogen may play an important role in the development and progression of papillary TC [45,46]. This might make it plausible that xenoestrogens, such as Cd and TCDD, may contribute to the risk of TC. Most papillary and follicular thyroid carcinomas are sporadic, but familial tumors may account for 5% of all TCs [47]. Several studies showed consistent positive associations between body mass index (BMI) and other indicators of adiposity (e.g., waist circumference and weight change) and TC [48,49]. The International Agency for Research on Cancer (IARC), in 2016, convened a working group to reassess the preventive effects of weight control on cancer risk. Based on the available data, the members of the working group concluded that there was sufficient evidence that the absence of excessive body fat lowered the risk of TC [50]. It is worth noting that some endocrine disruptors (e.g., PCBs, PBDE, cadmium, lead, and arsenic) are reported to be obesogenic [51,52], therefore, they might indirectly influence development or progression of TC. Some researchers have proposed that Hashimoto’s thyroiditis, an autoimmune disorder whose incidence is increased in the last decades, might predispose to papillary thyroid cancer (PTC). Several studies have found that the incidence of PTC is greater among patients with a diagnosis of Hashimoto’s thyroiditis. Some chemicals, such as heavy metals, chlorinated or brominated compounds and solvents, have the ability to induce autoimmunity, and to depress immune function [53]. Zaccarelli–Marino (2012) investigated incidences of Hashimoto’s thyroiditis in individuals living in the vicinity of petroleum industries, which may release PCDDs, PCDFs and several solvents into the environment [54]. She found an increase in incidence of Hashimoto’s thyroiditis in residents living near the petroleum industries, compared with residents living near a steel plant.

The aim of the present study was to estimate the incidence of TC, during the period from 2006 to 2013, by gender and age groups, in the population living in selected Italian National Priority Contaminated Sites (NPCS) with a documented presence of EDs considered thyroid carcinogens. The study is part of the epidemiological surveillance programme (Italian Epidemiological Study of Residents in National Contaminated Sites (SENTIERI Project)) carried out in NPCSs and promoted by the Italian Ministry of Health [41].

## 2. Materials and Methods

### 2.1. NPCSs Description

The NPCSs included in this study are, or were in the past, characterized by the presence of one or more polluting sources such as refineries, petrochemical and metallurgic plants, thermoelectric power plants, chemical plants, harbor areas, controlled and illegal waste dumps, and other industrial facilities. All these activities released into the environment several toxic chemicals, some of which with recognized or suspected endocrine disrupting activity. In all NPCSs, the industrial facilities operated or are still operating, in the vicinity or within urban areas for decades.

### 2.2. Study Design

TC incidence was investigated by gender and age-specific groups (adolescents and young adults aged 15–39 years, and adults aged 40 years and over) in populations living in the NPCSs, characterized by the presence of EDs considered potential thyroid carcinogens, during the study period 2006–2013 (with different time window lengths in the NPCSs studied).

Evidence of thyroid carcinogenicity of EDs was based on an a priori evaluation by the above-mentioned scientific institutions and advisory committees [17,18,19], as well as relevant literature published in the last eight years [20,21,22,23,24,25,26,27,28,29,30,31,32]. The search for literature published in the last eight years was performed using PubMed and Web of Science databases. Data on the presence of EDs in the NPCSs were derived from the legislative national decrees where the NPCSs are defined, in addition to local environmental agencies [55,56]. The available data are related to the presence of the substances in the various environmental matrices (soil, groundwater, and sea), not to their concentrations. By a search in PubMed, Web of Science, and other sources it was verified: (a) the existence of biomonitoring data reporting presence of high levels of EDs in residents in the NPCSs; and (b) other food and plants monitoring data [57,58,59,60,61,62,63,64,65,66,67,68,69,70,71,72].

**Figure 1:**
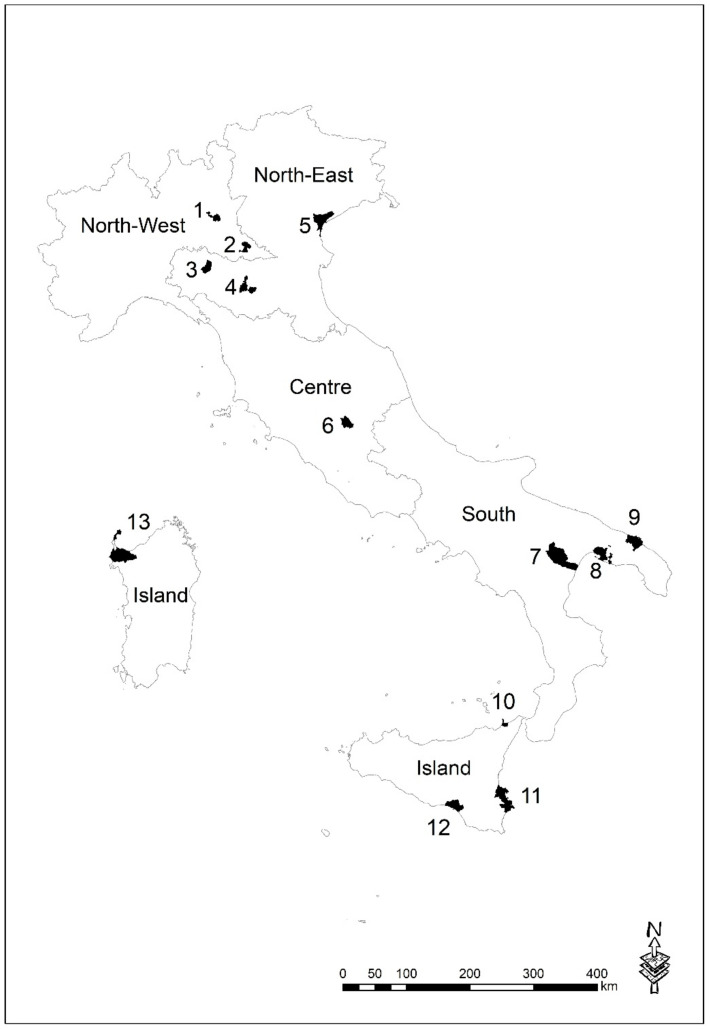
Italian geographical macro areas. National Priority Contaminated Sites (NPCS): 1 = Brescia-Caffaro; 2= Laghi di Mantova; 3 = Fidenza; 4 = Sassuolo; 5 = Venezia-Porto Marghera; 6 = Terni-Papigno; 7 = Area Industriale Val Basento; 8 = Taranto; 9 = Brindisi; 10 = Milazzo; 11 = Priolo; 12 = Gela; 13 = Area industriale Porto Torres.

Adjusted Standardized Incidence Ratios (SIRs), with their 90% confidence intervals (CIs), were computed by gender, and age-specific groups (aged 15–39 years, and 40 years or over) for each NPCS where a cancer registry participating with the SENTIERI Project was active and the information about municipality was available. This was accomplished by using the data from the database of the Italian Association of Cancer Registries (AIRTUM). For the computation of SIRs, the thyroid cancer cases observed in every NPCS were divided by the expected TC cases. The expected TC cases were obtained by using as reference the TC rates of the Italian macro area (North-West, North-East, Centre and South-Islands) (see Figure 1) where the NPCS is placed, as registered by the AIRTUM network. The thyroid cancer cases and the populations resident in the NPCSs were excluded from the computation of the reference rates. The reference population rates were computed by gender, and five-year age-class, referring to the 2006–2013 time window (with different time window lengths in the different NPCSs studied). SIRs were not calculated when fewer than five cases were observed considering the population of all ages by sex. The analyses were performed using the R statistical package, version 3.6.2 [73]. SIRs were not calculated for children, aged 0–14 years, due to the rarity of TC in this age group and to the fact that only very few cases were detected in the NPCSs. Furthermore, due to the low number of cases in some NPCSs it was not possible to calculate the SIRs for more age-specific groups.

The choice of the 90% significance level, according to the SENTIERI Project approach, was made to minimize the acritical use of CI as a surrogate of hypothesis testing; such use could lead to consider as relevant only the estimators for which the CI exclude the null value, i.e., the ones customarily defined as “statistically significant”. Especially when studying the risk from rare diseases or in vulnerable age and gender population subgroups it is more important to avoid a potential excess risk than to demonstrate a lack of risk. This is particularly relevant in epidemiological studies aimed at generating etiological hypothesis for public health purposes, while the size and precision of risk estimates can be fine-tuned, from a research perspective, in studies testing specific etiological hypotheses, where the standard 95% CI or even 99% CI are used.

## 3. Results

Sixteen NPCSs, served by a Cancer Registry and participating to the SENTIERI Project, were considered eligible for the study due to the presence of EDs reported to be potential TC carcinogens. Three NPCSs were excluded as information about municipality was not available to AIRTUM, therefore, a total number of thirteen NPCSs were included in the study. Ten out of the thirteen NPCSs were the subject of a previous study [38], regarding the period 1995–2006 (see above in Introduction).

Table 1 reports information on NPCSs pollution sources, years of start of first industrial activity, and EDs detected in environmental matrices, human biological samples, and food. Table 2 reports gender, and age-groups adjusted SIRs, with their 90% confidence intervals (90% CIs).

In the age group of 15–39 years, significantly increased SIRs for TC were found in two NPCSs, only in males (in these NPCSs an excess risk of TC was also found in both genders, in the age group of 40 years or over); a non-significant excess risk for TC was found in four NPCSs in males, and in five NPCSs in females. In the age group of 40 years and over, a significant excess risk of TC was found in six NPCSs in males and in seven NPCSs in females (the significant excesses were observed in five NPCSs in both genders); a non-significant increased risk was found in two NPCSs in both genders.

## 4. Discussion

The objective of this study was to investigate the possible relation between proximity to industrial facilities releasing EDs suspected to be thyroid carcinogens, and the incidence of TC. Some studies reported an increase in incidence or mortality of TC in population living in the proximity of industrial sites releasing EDs into the environment [35,36,37,38,39,40,41].

In our study, in the age group of 15–39 years, a statistically significant excess in incidence of TC was found only in two NPCSs (Sassuolo–Scandiano, and Terni–Papigno) in males, while non-significant excesses were found in four NPCSs in males and in five NPCSs in females. In twelve circumstances a significant deficit was reported. The low number of TC excesses in this age group might be explained, for most of the NPCSs, by the null or sparse number of cases on which the findings were based. In the age group of 40 years or over, a statistically significant excess in incidence of TC was found in six NPCSs (Brescia–Caffaro, Laghi Mantova, Milazzo, Sassuolo–Scandiano, Taranto, and Terni–Papigno) in males and in seven NPCSs (Brindisi, Brescia–Caffaro, Fidenza, Laghi Mantova, Sassuolo–Scandiano, Taranto, and Terni–Papigno) in females, while non-significant excesses were found in two NPCSs (Fidenza, and Area Industriale Porto Torres) in males and in three NPCSs (Gela, Milazzo, and Area Industriale Porto Torres) in females. In five NPCSs (Brescia–Caffaro, Laghi di Mantova, Sassuolo–Scandiano, Taranto, and Terni–Papigno) a statistically significant excess in incidence was found in both genders supporting a potential causal role of environmental exposure to EDs in the development of TC in these areas. In nine circumstances, in the same age group, a significant deficit was reported. However, the observed excesses were more frequent than the deficits.

In seven NPCSs, which were the subject of a previous study (Brescia Caffaro, Laghi di Mantova, Milazzo, Sassuolo–Scandiano, Taranto, and Terni Papigno) [38] in which SIRs were calculated only for all ages, several excesses in incidence of TC were found. In these NPCSs, excesses in incidence were also found in the present study, where SIRs were calculated by age-specific group. In the NPCS of Porto Torres, in which no excesses in incidence were observed in the previous study, non-significant increases in SIRs were found in males, in both age groups, in the present study.

The findings of several excesses in incidence, mainly observed in the age group of 40 years or over, are suggestive of a possible adverse effect associated with residence in NPCSs, even if a role of random variability, confounding and alternative explanations cannot be ruled out, due to the adoption of an ecological study design. However, this study restricted the analyses only to NPCSs where the environmental contamination by EDs possibly related to TC development was a priori documented. This reduced the possibility to observe spurious association only due to chance when looking to all different NPCSs.

A strength of this study is the availability of Cancer Registry data; this makes the case series complete, and with respect to the validity, complies with the requisites of the International Agency for Research on Cancer. Moreover, the SENTIERI project methodological approach used in this study has been recognized by the World Health Organization (WHO) as a first level approach to describe population health profiles in contaminated areas [74].

There are several limitations to our study, the most important of which is the lack of a quantitative indicator of residential exposure to EDs and the use of municipality as a minimum level of data aggregation. This reduces the possibility to further infer about causal associations. Another constraint is the low number of TC cases in most NPCSs in the age group of 15–39 years, and in some NPCSs in the age group of 40 years or over. Another limitation is the impossibility to account for other thyroid risk factors (e.g., obesity, family history of TC, occupational exposure to EDs, personal exposure to other types of EDs not considered in the present study (e.g., BPA, and flame retardants)).

Moreover, we could not estimate the magnitude of a possible overdiagnosis, since we had no information on the tumor size or cancer stage at diagnosis and whether populations living near NPCSs are more screened for TC. Thus, even if an increased incidence of TC was found in several NPCSs, the study design and the multifactorial etiology of thyroid cancer does not permit conclusions in terms of causal links with environmental contamination. Furthermore, the exposure to multiple chemicals with suspected endocrine disrupting properties, and the possibility of other mechanisms of their actions (oxidative stress, or DNA damage) [38], makes it difficult to hypothesize on substance and mechanisms that have determined the excesses of TC in some NPCSs.

However, the above-mentioned limitations typical of an ecological study design are at least partially balanced by the a priori selection of NPCSs with documented presence of EDs reported to possibly be carcinogenic to the thyroid.

## 5. Conclusions

As previously stated, the authors are aware of the limits of the used study design. However, beyond the usefulness of ecological studies for generating hypotheses, it should be taken into consideration that this approach made SENTIERI Project [43], on which this study is based, a permanent epidemiological surveillance system that provides Italy with official statistical data on NPCSs.

The novelty of this study was to investigate the incidence of TC in gender and age-specific groups in the NPCSs characterized by EDs environmental contamination. This approach identified possible population subgroups at higher risk of TC and provided indication of possible target population for future studies. To the best of our knowledge, this is the first study describing TC incidence by age-groups (both adults and young adults) in residents near industrially contaminated sites.

In this study, an excess risk of TC incidence was found in six NPCSs in males (46% of NPCSs) and in seven NPCSs in females (54% of NPCSs) in the age class of 40 years or over; the significant excesses were observed in both genders in five NPCSs (38% of NPCSs). Moreover, in two of these five NPCSs a significant excess risk of TC was also found in the age class of 15–39 years, only in males. These results are overall suggestive of a potential etiological role of residential exposure to EDs in the development of TC, mainly in the age class of 40 years or over, in the communities living in these areas.

The observation of several excesses of TC incidence encourages further analytical epidemiological studies to be performed. Future analytical studies, with robust sample size, should attempt to collect information on occupational and residential history, obesity, TC family history, diagnosis of Hashimoto’s thyroiditis, and on TC tumor histotypes and possibly tumor size. Considering that the effects of some EDs (e.g., TCDD, cadmium, and lead) may progress even after exposure reduction, continuing epidemiological surveillance of residents in the NPCSs studied is recommended. Epidemiological surveillance should also include a biomonitoring programme, to determine levels of EDs in residents living in the NPCSs.

## Figures and Tables

**Table 1:** National Priority Contaminated Sites (NPCS) information on pollution sources, year of start of first industrial activity, endocrine disruptors (EDs) of interest detected in environmental matrices, human biological samples, and food.

NPCS	Pollution Source	Start of First Industrial Activity (Year)	EDs of Interest Detected in Environmental Matrices [55,56]	Human Biomonitoring	EDs Detected in Food
Area industriale Val Basento (Southern Italy)	Chemical plant	1961	Cd, Hg, solvents		
Brindisi (Southern Italy)	Chemical plant, petrochemical plant, oil refinery, electric power plant, harbor area, industrial waste landfill	1959	Cd, Pb, As, PCB, chlorobenzene, other solvents		
Brescia Caffaro * (North West Italy)	Chemical plants, landfill	1930	As, PCBs, PCDDs/PCDFs, chlorobenzene	PCDDs/PCDFs, PCBs (serum) [57,58]	PCB (food of animal and vegetal origin);PCDDs/PCDFs, PCB (cattle’s meat, cow milk, forage) [59]
Fidenza * (North East Italy)	Chemical plants, urban and hazardous waste landfills	1888	As, PCBs, PCDDs, benzene, other solvents		
Gela (Southern Island)	Chemical plant, Petrochemical plant, oil refinery, industrial waste landfill	1965	As, PCB, benzene, other solvents,	As, Hb, Pb (urine, serum); 59 PCBs congeners (serum) [60]	
Laghi Mantova * (North West Italy)	Metallurgy plants, paper plant, petrochemical plant, harbor area, industrial waste landfills, hazardous waste incinerator	1953	AS, Cd, PCDDs, ethylbenzene, other solvents	PCDDs, PCBs (serum) [61]	PCBs (fruit, vegetables) [62]
Milazzo * (Southern Italy)	Oil refinery, steel plant, thermal power plant, electrical equipment factories, illegal dumping site	1961	PCDDs, heavy metals. Benzo(a)pyrene	As, Cd, Hg (urine); Pb (serum) [63]	
Area industriale Porto Torres * (Southern Island)	Chemical plants, petrochemical plant, oil refinery, power plant, harbor area, illegal dumping site	1962	As, Cd, chlorobenzene, other solvents		
Priolo * (Southern Island)	Chemical plants, petrochemical plant, refinery, harbor area, hazardous waste landfills	1949	PCB, hexachlorobenzene		As, Cd, Pb, Hg, PHSs organochlorine compounds (meat, milk, seafood) [64]
Sassuolo–Scandiano * (North East Italy)	Ceramic industries, industrial waste landfills	1920	Heavy metals		
Taranto * (Southern Italy)	Oil refinery, steel plant, harbor area, cement plant, controlled and illegal waste dumps	1945	As, Cd, PCDDs, PCBs, benzene, xylene	As, Cd (serum, urine): PCDDs, PCBs (serum, milk) [65,66,67,68,69]	PCDDs, PCBs (sheep and cow’s milk, clams); PCB, HCB, PAHs (clams) [70,71]
Terni–Papigno * (Central Italy)	Steel plant, hazardous waste landfills	1935	PCB		
Venezia Porto Marghera * (North East Italy)	Chemical plants, petrochemical plant, oil refinery, harbor area, illegal dumping sites	1924	As, Cd, PCBs, PCDDs, solvents		As, Cd, PCDDs, PCDFs (shellfish) [72]

* NPCS included in Benedetti et al. (2017) previous study [38]; Abbreviations: NPCS: National Priority Contaminated site; As: Arsenic; Cd: Cadmium; BPA: Bisphenol A; EDs: Endocrine disruptors; HCB: Hexachlorobenzene; Hg: mercury; PAHs: Polycyclic Aromatic Hydrocarbons; Pb: lead; PCBs: polychlorinated biphenyls; PCDDs: Polychlorinated dibenzo-p-dioxins; PCDFs: Polychlorinated dibenzofurans.

**Table 2:** Thyroid cancer Standardized Incidence Ratios (SIRs) with 90% confidence intervals (CIs) by gender, and age group in selected NPCSs with documented presence of EDs.

NPCS	Age Group of 15–39 Years						Age gGroup of 40 Years or Over						Referred to the Years
	Males			Females			Males			Females			
	Population	Obs.	SIRs (90% CI)	Population	Obs.	SIRs (90% CI)	Population	Obs.	SIRs (90% CI)	Population	Obs.	SIRs (90% CI)	
Area industriale Val Basento (Southern Italy)	41,080	0	-	39,899	<3	NA	57,478	5	78 (37–163)	64,456	17	62 (42–93)	2006–2010
Brindisi (Southern Italy)	46,417	3	95(37–244)	46,609	9	77 (44–132)	62,798	8	94 (53–169)	74,289	38	134 (102–175)	2006–2008
Brescia Caffaro * (North West Italy)	92,518	7	146 (79–272)	88,319	18	128 (87–189)	156,289	30	170 (126–230)	193,772	80	152 (126–182)	2006–2008
Fidenza * (North East Italy)	52,711	3	86 (33–222)	50,531	16	140 (93–211)	97,675	20	132 (92–191)	126,580	63	153 (124–188)	2006–2013
Gela (Southern Island)	54,855	4	74 (33–169)	55,971	21	105 (73–150)	67,558	8	59 (32–104)	73,461	45	104 (81–133)	2007–2012
Laghi di Mantova * (North West Italy)	42,121	0	-	39,984	6	94 (48–184)	77,992	16	196 (130–295)	100,511	41	161 (124–208)	2006–2010
Milazzo * (Southern Island))	54,544	6	173 (88–339)	53,104	10	81 (48–136)	79,576	16	168 (111–254)	91,377	36	119 (90–157)	2006–2011
Area industriale Porto Torres * (Southern Island)	153,285	13	119 (75–188)	149,155	31	78 (58–104)	226,528	35	114 (87–151)	264,859	106	103 (88–121)	2006–2011
Priolo * (Southern Italy)	221,991	11	68 (42–112)	215,146	43	75 (59–97)	309,143	33	76 (57–101)	345,748	105	77 (66–91)	2006–2012
Sassuolo–Scandiano * (North East Italy)	136,530	18	202 (137–297)	129,176	30	104 (77–141)	201,585	54	175 (140–219)	217,998	125	153 (132–177)	2006–2012
Taranto * (Southern Italy)	244,733	22	135 (95–191)	241,659	68	113 (92–138)	349,441	87	193 (162–230)	424,559	215	139 (125–156)	2006–2012
Terni–Papigno * (Central Italy)	124,927	14	159 (102–247)	126,072	17	54 (36–80)	238,796	49	149 (117–188)	290,818	112	119 (102–139)	2006–2013
Venezia * (North East Italy)	147,945	9	91 (53–157)	138,601	11	34 (21–56)	298,547	35	75 (57–99)	369,088	80	60 (50–72)	2006–2009

* NPCS included in Benedetti et al. (2017) previous study [38]; NPCS: National Priority Contaminated Site; EDs: endocrine disruptors; Obs.: observed; NA: Not Analyzed, as the number of cases was less than three

## Data Availability

The data presented in this study are available on request from the corresponding author. The data are not publicly available due to ethical and privacy issues.

## Abbreviations

The following abbreviations are used in the manuscript:

AIRTUM	Italian Association of Cancer Registries
As	Arsenic
Cd	Cadmium
CI	Confidence Interval
COD	Chemical Oxygen Demand
BPA	Bisphenol A
EDs	Endocrine disruptors
IARC	International Agency for Research on cancer
HCB	Hexachlorobenzene
NPCSs	National Priority Contaminated Sites
PAHs	Polycyclic Aromatic Hydrocarbons
PBDEs	Polybrominated Diphenyl Ethers
PCBs	Polychlorinated Biphenyls
PCDDs	Polychlorinated Dibenzo-p-dioxins
PCDFs	Polychlorinated Dibenzofurans
Pb	Lead
SIRs	Standardized Incidence Ratios
TC	Thyroid cancer
TCDD	2,3,7,8-Tetrachlorodibenzo-p-dioxin
WHO	World Health Organization

## Appendix A

AIRTUM Working Group

Name	Affiliation
Antonino Ardizzone	Registro tumori Brindisi
Adele Caldarella	Registro tumori toscano, Istituto per lo studio, la prevenzione e la rete oncologica (ISPRO), Firenze
Lorenza Boschetti	Registro tumori Pavia
Angelita Brustolin	Registro tumori Viterbo c/o Dipartimento prevenzione ASL Viterbo
Rossella Cavallo	Registro tumori Salerno
Giuseppina Candela	Registro tumori Trapani
Giuliano Carrozzi	Registro tumori Modena
Luca Cavalieri D’Oro	Registro tumori ATS della Brianza
Rosaria Cesareccio	Registro tumori Sassari
Giorgio Chiaranda	Registro tumori Piacenza
Maria Lia Contrino	Registro tumori integrato Catania-Messina-Siracusa-Enna
Antonella Dal Cin	Registro tumori Veneto
Fabio Falcini	Registro tumori Romagna
Anna Clara Fanetti	Registro tumori Sondrio
Stefano Ferretti	Registro tumori Ferrara
Rosa Filiberti	Registro tumori Genova
Rocco Galasso	Registro tumori Basilicata
Iolanda Grappasonni	Registro tumori infantili Marche c/o Università di Camerino
Silvia Iaccovacci	Registro tumori Latina
Michele Magoni	Registro tumori Brescia
Lucia Mangone	Registro tumori Reggio Emilia
Guido Mazzoleni	Registro tumori Alto Adige
Anna Melcarne	Registro tumori Lecce
Maria Michiara	Registro tumori Parma
Aldo Minerba	Registro tumori Taranto
Fernando Palma	Registro tumori Foggia
Silvano Piffer	Registro tumori della provincia di Trento c/o Azienda provinciale servizi sanitari, Provincia di Trento
Paolo Ricci	Registro tumori Mantova
Stefano Rosso	Registro tumori del Piemonte
Antonio Giampiero Russo	Registro tumori ATS della Città Metropolitana di Milano
Carlotta Sacerdote	Registro tumori infantili Piemonte
Giuseppe Sampietro	Registro tumori della ATS di Bergamo
Salvatore Sciacca	Registro tumori integrato Catania-Messina-Siracusa-Enna
Antonella Sutera	Registro tumori Catanzaro
Giovanna Tagliabue	Registro tumori di Varese, Fondazione IRCCS Istituto Nazionale dei Tumori
Rosario Tumino	Registro Tumori Ragusa-Caltanissetta, ASP 7 Ragusa
Mario Usala	Registro tumori Nuoro
Francesco Vitale	Registro tumori Palermo e provincia c/o UOC epidemiologia clinica, Azienda ospedaliero-universitaria Policlinico P. Giaccone, Palermo
Registro Tumori Barletta	Contributed by providing cancer registry data for the calculation of reference rates
